# The p.Ser107Leu in *BICD2* is a mutation ‘hot spot’ causing distal spinal muscular atrophy

**DOI:** 10.1093/brain/awv159

**Published:** 2015-06-10

**Authors:** Boglarka Bansagi, Helen Griffin, Venkateswaran Ramesh, Jennifer Duff, Angela Pyle, Patrick F. Chinnery, Rita Horvath

**Affiliations:** 1 The John Walton Muscular Dystrophy Research Centre, MRC Centre for Neuromuscular Diseases, Institute of Genetic Medicine, Newcastle University, Newcastle upon Tyne, UK; 2 Wellcome Trust Centre for Mitochondrial Research, Institute of Genetic Medicine, Newcastle University, Newcastle upon Tyne, UK; 3 Department of Paediatric Neurology, Royal Victoria Infirmary, Newcastle upon Tyne Foundation Hospitals NHS Trust, Newcastle upon Tyne, UK

Sir,

We read with great interest the recent paper by Rossor *et al.* (2015), reporting two new patients and summarizing observations on 30 previously published patients worldwide with *BICD2*-related spinal muscular atrophy. The work provides a timely overview of the main clinical characteristics observed in patients carrying the six currently known dominant *BICD2* mutations. The paper also highlights the current knowledge of the structural and interaction specificities of BICD2 protein affected by the described missense mutations and supports dynein-dynactin trafficking impairment as a common pathomechanism (Oates *et al.*, 2013).

The most common *BICD2* mutation reported in four of the nine families was c.320C>T, p.Ser107Leu. The mutation in two large pedigrees originating from Australia and Austria fell on a shared eight single nucleotide polymorphism (SNP) haplotype, spanning a 0.1 Mb region and with a background frequency of 2% in the European population, suggesting a recent founder mutation between the two families. The same haplotype background was predicted in another affected family from the USA with European ancestors. A four-generation Bulgarian family with Turkish ethnic origin, originally reported by Peeters *et al.* (2013), was the fourth family carrying the same mutation within a 12.7 Mb region of linkage. In this family the disease arose as a consequence of a *de novo* mutation in the proband. In addition a three-generation Dutch family published by Neveling *et al.* (2013), had a similar phenotype and carried the same c.320C>T, p.Ser107Leu missense mutation within a 10 Mb region of linkage.

Here, we would like to expand the clinical presentation and haplotype background of the p.Ser107Leu *BICD2* mutation with five additional patients from two North UK families (Fig. 1). A 41-year-old mother presented with her three children affected by the same disorder (Family 1). She had a longstanding history of muscle weakness that has remained stable throughout her life. She was born with toe deformities and had mildly delayed motor development. She always had difficulties rising from the floor and with physical exercise. She developed a locked jaw in her university years and she was provided with a night splint to prevent its recurrence. On neurology examination she had mild weakness both proximally and distally in her lower limbs. She had a positive Gower’s manoeuvre and she could not perform heel walking. Muscle strength in her upper limbs was entirely preserved but there was a mild scapular winging on the left side. Her deep tendon reflexes were brisk and asymmetric in her upper limbs. No cranial nerve involvement and sensory impairment were found. Except for a mildly elevated creatine kinase (292 U/l, normal <200), all other laboratory parameters were normal. Sensorimotor nerve conduction velocities and action potentials were within the normal ranges. On electromyography she had evidence of severe chronic neurogenic changes in both her upper and lower limbs. The findings were compatible with segmental motor neuronopathy. Her muscle MRI showed symmetrical prominent fatty replacement of gluteus medius, vastus lateralis, rectus femoris, semi-membranosus, lateral and medial gastrocnemius.

**Figure 1:**
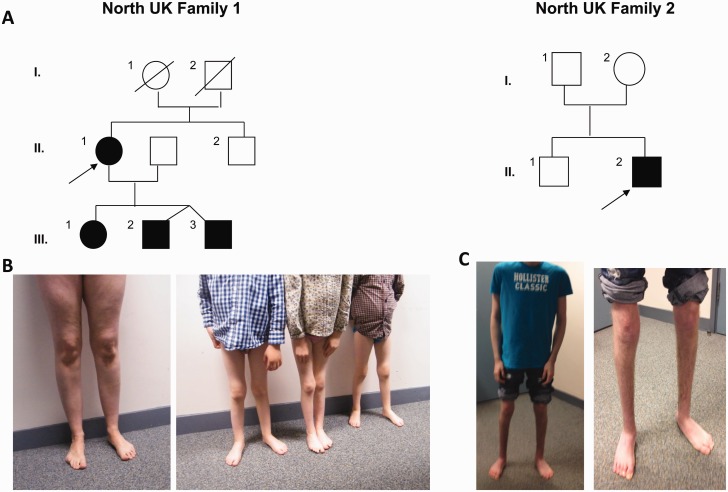
**Pedigrees and clinical presentations of 2 new families carrying the p.Ser107Leu mutation in *BICD2***. (**A**) Pedigrees of the North UK families. Probands of each family are indicated by an arrow. (**B**) Image of the proband and her three affected children from Family 1 presenting with lower limb wasting and feet deformities. (**C**) Images of proband from Family 2 demonstrating wasting of lower extremities and his postsurgical feet position.

Her pregnancies and deliveries were uncomplicated. Her first born, currently 6-year-old daughter was noted to have a minor foot abnormality at birth. She did not walk until 20 months of age, and started walking on the medial aspect of her feet. The 5-year-old twin brothers had a very similar clinical course. Both had mild motor delay, and currently have mild weakness in both proximal and distal muscles of their lower limbs associated with disproportionate atrophy of the distal muscles (Fig. 1). Their upper body and arms are particularly strong with good manual dexterity but they show mild scapular winging. All three children have difficulties with rising from the floor, climbing stairs, running and they cannot perform heel walking. All receive physiotherapy and orthotic support. All four family members have normal cognition.

Another North British individual patient had no family history of neuromuscular disease (Family 2). His parents were non-consanguineous and healthy, and his older brother is asymptomatic. The proband was born from an unremarkable pregnancy and delivery, but at birth he was noted with congenital bilateral talipes. After trials of physiotherapy and serial castings of his foot, the deformity was surgically corrected at age 1 year. His motor development was subsequently delayed, and he started walking only around the age of 3 years. He remained ambulant with a waddling gait, but he requires wheelchair support for longer distances. His cognition is normal.

On examination he had mild weakness, predominantly in the proximal muscles of his lower limbs accompanied by marked atrophy in the hip muscles. There was prominent muscle amyotrophy of both anterior and posterior compartments below the knee. He had full muscle power in the shoulders and upper limbs but he showed scapular winging bilaterally. Upper limb reflexes were easy to elicit while lower limb reflexes were reduced. He walked with a high-steppage gait and he could not achieve heel strike. Sensation was preserved. Nerve conduction studies were normal. Electromyography revealed fibrillation potentials and positive sharp waves and motor units were of markedly increased amplitude, polyphasic and unstable, suggesting chronic neurogenic process with spinal motor neuron involvement. Muscle biopsy showed neurogenic fibre atrophy but no other specific changes.

The clinical presentation of these five new patients is similar to the patients reported by Rossor *et al.* (2015); however, we would emphasize that all our patients showed scapular winging, which we believe is a common feature of BICD2 deficiency. The heterozygous c.320C>T, p.Ser107Leu mutation is the cause of the disease in both families. It followed an autosomal dominant inheritance pattern in Family 1, however it is *de novo* in Family 2, as both parents were tested negative. It is indicating that the c.320C>T, p.Ser107Leu mutation arose independently in these two families. This mutation is located in a methylated CpG dinucleotide suggesting that recurrent mutations may occur at this position due to methylation-mediated deamination.

To determine whether the c.320C>T, p.Ser107Leu mutation in Family 1 is present on the same haplotype background as three of the families reported by Rossor *et al.* (2015) or rather is a mutational hot spot, we performed haplotype analysis. HapMap (data release 28) SNP genotype data from the CEU population, in a region surrounding *BICD2* were used to generate Haplotypes in Haploview (www.broadinstitute.org). There were 10 different haplotypes present in the CEU European population in a single 0.1 Mb block (Fig. 2). Genotypes from whole exome sequence data were present for the first five SNPs of the haplotypes and genotyping of a further seven haplotype tagging SNPs allowed us to show that the mutation was carried on a different haplotype background each of our two families. Additionally, the mutation-carrying haplotype in Family 1 was present in 9.8% of the HapMap European population, which is presumably different from the haplotype reported by Rossor *et al.* (2015), which was present in only 2% of Europeans. Our haplotype analysis excludes the presence of a common founder mutation and further supports the theory that the c.320C>T position is as a mutational hot spot.

**Figure 2:**
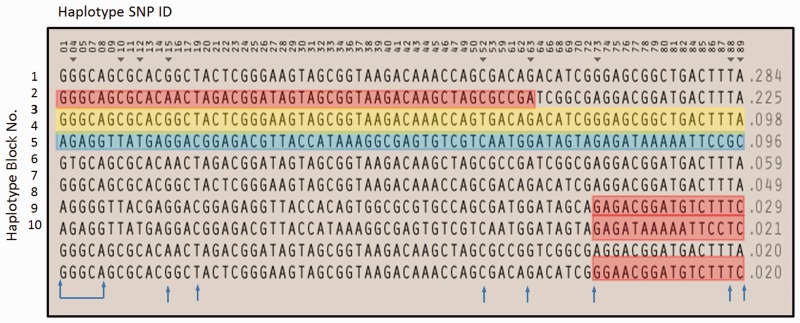
**Ten European haplotype blocks in a 0.1Mb region surrounding *BICD2* from Haplotype SNP IDs 01 (rs556) to 89 (rs10117449).** The *BICD2* c.320C>T mutation is located between SNPs 19 and 20. Haplotype SNPs 01, 04, 05, 07 and 08 were genotyped from whole exome sequence; SNPs 15, 19, 52, 63, 73, 88 and 89 were genotyped from Sanger sequence (blue arrows). Affected members of Family 1 shared haplotype block 3 (yellow box), present in the HapMap CEU population at a frequency of 0.098. The affected proband in Family 2 had haplotype blocks 2 and 4 (red and blue boxes) in the region surrounding the mutation; recombination not observed in the CEU population occurred between SNPs 63 and 73, so that this proband also had part of either blocks 7, 8 or 10.

The detection of more *de novo* cases suggests that the diagnosis of *BICD2* mutations should be considered even with no positive family history, and particularly when there is scapular winging associated with early onset distal lower limb motor neuropathy.

## Funding

B.B. is supported by the MRC Centre for Neuromuscular Diseases. R.H. is supported by the Medical Research Council (UK) (G1000848) and the European Research Council (309548). P.F.C. is a Wellcome Trust Senior Fellow in Clinical Science and an NIHR Senior Investigator who also receives funding from the Medical Research Council (UK), the UK Parkinson’s Disease Society, and the UK NIHR Biomedical Research Centre for Ageing and Age-related disease award to the Newcastle upon Tyne Foundation Hospitals NHS Trust.

## References

[awv159-B1] Neveling K Martinez-Carrera LA Hölker I Heister A Verrips A Hosseini-Barkooie SM Mutations in BICD2, which encodes a golgin and important motor adaptor, cause congenital autosomal-dominant spinal muscular atrophy. Am J Hum Genet 2013; 92: 946–54. 23664116 10.1016/j.ajhg.2013.04.011 PMC3675237

[awv159-B2] Oates EC Rossor AM Hafezparast M Gonzalez M Speziani F MacArthur DG Mutations in BICD2 cause dominant congenital spinal muscular atrophy and hereditary spastic paraplegia. Am J Hum Genet 2013; 92: 965–73. 23664120 10.1016/j.ajhg.2013.04.018 PMC3675232

[awv159-B3] Peeters K Litvinenko I Asselbergh B Almeida-Souza L Chamova T Geuens T Molecular defects in the motor adaptor BICD2 cause proximal spinal muscular atrophy with autosomal-dominant inheritance. Am J Hum Genet 2013; 92: 955–64. 23664119 10.1016/j.ajhg.2013.04.013 PMC3675262

[awv159-B4] Rossor AM Oates EC Salter HK Liu Y Murphy SM Schule R Phenotypic and molecular insights into spinal muscular atrophy due to mutations in BICD2. Brain 2015; 138: 293–310. 25497877 10.1093/brain/awu356 PMC4306822

